# Fuzzy Approximate Entropy of Extrema Based on Multiple Moving Averages as a Novel Approach in Obstructive Sleep Apnea Screening

**DOI:** 10.1109/JTEHM.2022.3197084

**Published:** 2022-08-11

**Authors:** Peiyu Weng, Keming Wei, Tian Chen, Mingjing Chen, Guanzheng Liu

**Affiliations:** Key Laboratory of Sensing Technology and Biomedical Instrument of Guangdong Province, School of Biomedical EngineeringSun Yat-sen University 26469 Guangzhou 510006 China

**Keywords:** Obstructive sleep apnea (OSA), autonomic nervous system (ANS), heart rate variability (HRV), apnea-hypopnea index (AHI), fuzzy approximate entropy of extrema based on multiple moving averages (Emma-fApEn)

## Abstract

Objective: Obstructive sleep apnea (OSA) is a respiratory disease associated with autonomic nervous system dysfunction. As a novel method for analyzing OSA depending on heart rate variability, fuzzy approximate entropy of extrema based on multiple moving averages (Emma-fApEn) can effectively assess the sympathetic tension limits, thereby realizing a good performance in the disease severity screening. Method: Sixty 6-h electrocardiogram recordings (20 healthy, 16 mild/moderate OSA and 34 severe OSA) from the PhysioNet database were used in this study. The performances of minima of Emma-fApEn (fApEn-minima), maxima of Emma-fApEn (fApEn-maxima) and classic time-frequency domain indices for each recording were assessed by significance analysis, correlation analysis, parameter optimization and OSA screening. Results: fApEn-minima and fApEn-maxima had significant differences between the severe OSA group and the other two groups, while the mean value (Mean) and the ratio of low-frequency power and high-frequency power (LH) could significantly differentiate OSA recordings from healthy recordings. The correlation coefficient between fApEn-minima and apnea-hypopnea index was the highest (|R| = 0.705). Machine learning methods were used to evaluate the performances of the above four indices. Random forest (RF) achieved the highest accuracy of 96.67% in OSA screening and 91.67% in severe OSA screening, with a good balance in both. Conclusion: Emma-fApEn may be used as a simple preliminary detection tool to assess the severity of OSA prior to polysomnography analysis.

## I. Introduction

Obstructive sleep apnea (OSA) is a chronic and curable upper respiratory tract disease, characterized by repeated blockage of the upper respiratory tract during sleep, which will lead to poor quality of sleep and thus daytime sleepiness [1]. The occurrence mechanism of OSA includes upper airway abnormalities [2], significant depressions of the neuromuscular compensatory responses [3], [4], low arousal thresholds [5], etc. It’s reported that OSA is closely associated with diabetes [6], atherosclerosis [7], stroke [8], hypertension [9] and a variety of cardiovascular diseases [10]. The severity of OSA can also be used as a predictor of mortality [11]. At present, there are at least about 1 billion OSA patients in the world [12], and the prevalence of OSA is showing an upward trend [13]. Moreover, many OSA patients are still undiagnosed [14]. Therefore, there is an urgent need for a simple and effective method of detecting OSA.

Polysomnography is considered as a gold standard for OSA detection [15]. However, it has some disadvantages including being expensive, causing patients’ discomfort, requiring expert judgment and having obscure results [16]. In recent years, many researchers have proposed more efficient methods for OSA detection. OSA can affect a patient’s breathing state, nerve activity, muscle activity, heart function and so on. Consequently, some studies developed OSA detection algorithms based on oxygen saturation (SpO_2_) [17], [18], [19], [20], electroencephalogram (EEG) [21], [22], [23], [24], electromyography (EMG) [25], [26], [27], electrocardiogram (ECG) [28], [29], [30], [31], etc. The results of these studies are relatively easy to interpret and do not require expert judgment. Since the heart rate of the patients often changes during OSA attacks [32], heart rate variability (HRV) analysis, which can be derived from ECG signals, is one of the simplest and most effective methods for detecting OSA.

HRV reflects slight changes in the heartbeat cycle. It is produced by the interaction between autonomic nervous system (ANS) and internal nervous system of the heart [33]. Sympathetic nerve activity has been proved to be affected by apneic events [34]. As a result, as a non-invasive tool, HRV analysis provides an effective evaluation of ANS function [35], and can be used to assess the integrity of ANS [36]. The classic HRV analysis includes time and frequency domain analysis. Roche *et al.* [37] reported that the time domain HRV indices were significantly associated with the diseased status, which achieved a sensitivity of 90% in OSA diagnosis. Gula *et al.* [38] proved the practicability of the frequency domain HRV indices in OSA discrimination, and suggested that the ratio of low-frequency power and high-frequency power (LH) was the most useful index for OSA detection. Though LH has a great performance in HRV analysis, it only reflects the ANS fluctuation in a certain period.

However, many linear indices of HRV are susceptible to the heart rate spontaneous fluctuation [39], [40]. Moreover, the linear indices of HRV are not enough to characterize the complex dynamics of heartbeat [41]. In recent years, many researchers began to use nonlinear analysis methods in OSA detection. Entropy is a common index quantifying nonlinear dynamics, which can be used to assess the complexity of HRV. Haitham *et al.* [42] measured HRV based on sample entropy and found that the complexity of HRV was significantly different between normal people and OSA patients. Liang *et al.* [43] proposed nonparametric sample entropy, which can evaluate and quantify the severity of OSA patients. Ravelo-García *et al.* [44] reported that permutation entropy could effectively detect OSA patterns in heart rate. Li *et al.* [45] used variance delay fuzzy approximate entropy to distinguish the OSA patients and achieved the correct grouping of any two groups of the normal, mild/moderate OSA and severe OSA. The above studies supported that the nonlinear method could discover the abnormality of ANS in OSA patients.

The performance of OSA classification is not only affected by features, but also related to the quality of the classifier. Janbakhshi *et al.* [46] proposed an ECG method to identify OSA, and combined it with support vector machine (SVM), k-nearest neighbor, linear discriminant and quadratic discriminant methods to extract RR interphase features from ECG-derived respiration sequences, achieving 90.9% accuracy. Sharma *et al.* [47] used a linear combination of low-order Hermite basis functions to simulate QRS ECG signals, and used a least squares SVM classifier with Gaussian radial basis function performance as a radial basis function to classify apnea events. Alireza *et al.* [48] proposed a random forest classifier to classify features after dimensionality reduction by principal component analysis and linear discriminant analysis, achieving an accuracy of 95.01%.

In above studies, most of them were analyzed by comparing the indices of the overall data of OSA groups and control groups, and less attention was paid to the information of some special points in the data. In HRV analysis, the general trend of the RR interval sequences (RRs) reflects the general changes in the ANS tension, and the extrema of the RRs are related to the ANS tension limits.

Therefore, in order to analyze the changes of the ANS tension limits in OSA patients through the extrema in the general trend of the RRs, this study proposed the fuzzy approximate entropy of extrema based on multiple moving averages (Emma-fApEn). Due to the small partial fluctuations of the RRs, the multiple moving average method was introduced into the experiment, which reduces the fluctuation effect of the sequence by obtaining segments’ averages of the data several times. Sample entropy has the advantages of anti-noise and anti-interference, but it depends on small tolerances and forward matching of data length [49]. Permutation entropy can reflect the regularity of time series, but it is not sensitive to the outliers in internal series [50]. Fuzzy approximate entropy (fApEn) shows better monotonicity, consistency and robustness when describing signals of different complexities [51]. So fApEn was applied to represent the complexity of extrema. Moreover, the Emma-fApEn method was combined with random forest (RF) classifier, to achieve OSA screening. Therefore, the multiple moving average method combined with the standardization method was used to alleviate the small partial self-fluctuation of RRs. Then the extremum sequences reflecting ANS tension limits were extracted. By applying fApEn, Emma-fApEn was obtained from extremum sequences. To evaluate the complexity of fluctuations of the ANS tension limits, we conducted the significance analysis, the correlation analysis, the parameter optimization, and the OSA screening for Emma-fApEn.

## II. Method

### A. Data

The database used in this paper was downloaded from https://www.physionet.org/physiobank/database/apnea-ecg/, which was applied in the Computers in Cardiology Challenge 2000 [52]. The database includes 70 single-channel nocturnal ECG recordings from 32 OSA patients and normal subjects. In this database, the sampling frequency of each recording is 100Hz, and the recording time is 401~587min. Each minute of each recording has a corresponding reference note, which identifies whether apnea occurs in that minute.

The number of apnea and hypopnea events per hour during sleep is defined as the apnea-hypopnea index (AHI), which is an indicator of the severity of sleep apnea. Recordings that contained at least 1 hour with AHI of 10 or more, and at least 100 minutes labeled apnea were classified as the apnea group. Recordings that contained at least 1 hour with AHI of 5 or more, and between 5 and 99 minutes labeled apnea were classified as the borderline group. Recordings that contained at least 1 hour with AHI of less than 5, and fewer than 5 minutes labeled apnea were classified as the control group. In this study, the apnea and control groups were used for analysis. According to AHI, the selected recordings were divided into the normal group (20 recordings, AHI<5), the mild/moderate OSA group (14 recordings, 5≤AHI<30), and the severe OSA group (26 recordings, AHI≥30).

### B. HRV Analysis Method

**FIGURE 1.**
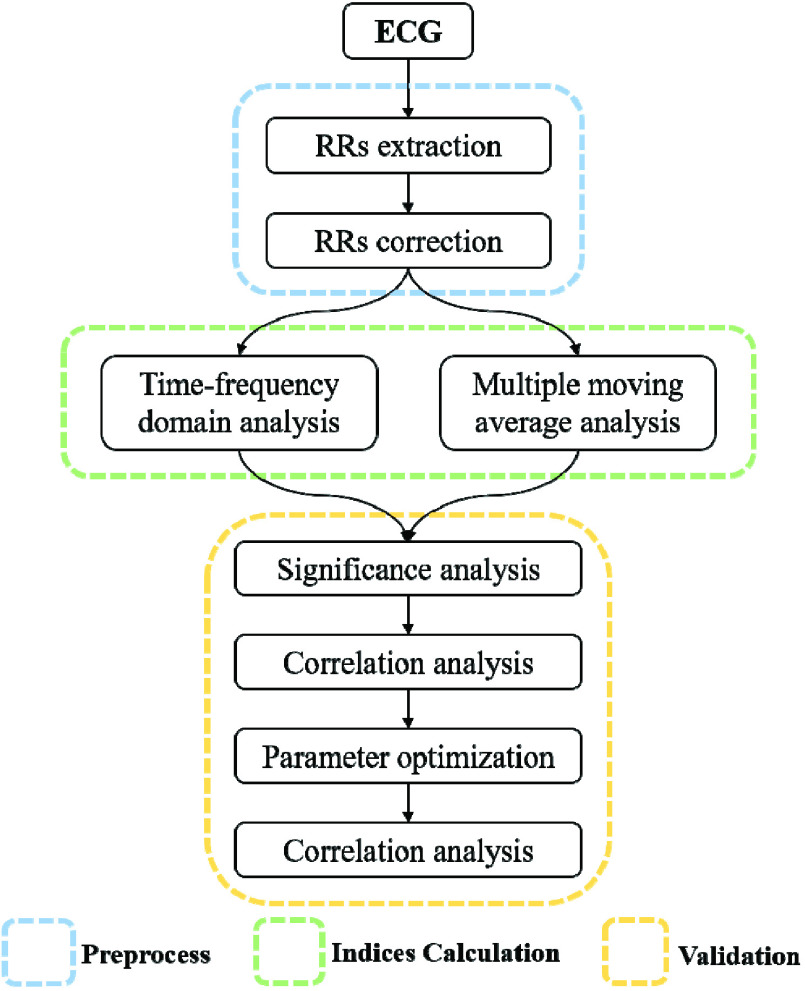
The general frame diagram of the HRV analysis method.

The HRV analysis method used in this study is shown in Fig. 1. First, the RRs were extracted and corrected. Then, the time-frequency domain indices and multiple moving average indices of the RRs were calculated. Finally, the effectiveness of the above indices was verified and analyzed through the significance analysis, the correlation analysis, the parameter optimization and the machine learning classifications.

#### 1) Preprocess

First, the segments with large errors in the beginning and end of the recordings were cut, and then the first 6 hours of the recordings were selected as the ECG signals for the study. Next, the RRs from the ECG signal were extracted according to the Pan-Tompkins algorithm [53]. Finally, an improved local median filter was used to eliminate the unexpected data spikes in the RRs [54].

#### 2) Time Domain Indices

HRV is the small difference in timing between successive normal (sinus) cardiac cycles. We completed the time domain analysis by the time domain measurement of HRV. The common time domain indices for HRV analysis of OSA include the mean value (Mean), the standard deviation of the normal-to-normal intervals (SDNN), and the square root of the mean of the sum of the squares of differences between adjacent the normal-to-normal intervals (RMSSD) of consecutive 5-minute RR intervals. For N points RRs consisting of consecutive 5-minute RR intervals, they are defined as follows:

\begin{align*} Mean=&\frac {1}{N}\sum \limits _{i=1}^{N} {RRs_{i}} \tag{1}\\ SDNN=&\sqrt {\frac {1}{N}\sum \limits _{i=1}^{N} {\left({RRs_{i} -\frac {1}{N}\sum \limits _{i=1}^{N} {RRs_{i}} }\right)^{2}}} \tag{2}\\ RMSSD=&\sqrt {\frac {1}{N-1}\sum \limits _{i=1}^{N-1} {(RRs_{i+1} -RRs_{i})^{2}}}\tag{3}\end{align*}

#### 3) Frequency Domain Indices

The frequency domain analysis is to analyze the law of heart rate change based on the power spectral density of RRs calculated by fast Fourier transform. It is relevant to time domain analysis, and moreover, it can reveal the more complex changes of heart rate. Frequency domain indices commonly used for HRV analysis of OSA patients include low-frequency power (LF, 0.04–0.15Hz), high-frequency power (HF, 0.15–0.4Hz) and LF/HF (LH) for 5-minute epochs. The formulas are as follows:

\begin{align*} LF=&\int _{0.04\times 2\pi }^{0.15\times 2\pi } {\vert F_{\omega } \vert ^{2}d\omega } \tag{4}\\ HF=&\int _{0.15\times 2\pi }^{0.4\times 2\pi } {\vert F_{\omega } \vert ^{2}d\omega } \tag{5}\\ LH=&\frac {LF}{HF}\tag{6}\end{align*}

#### 4) Indices of Emma-fApEn

**FIGURE 2.**
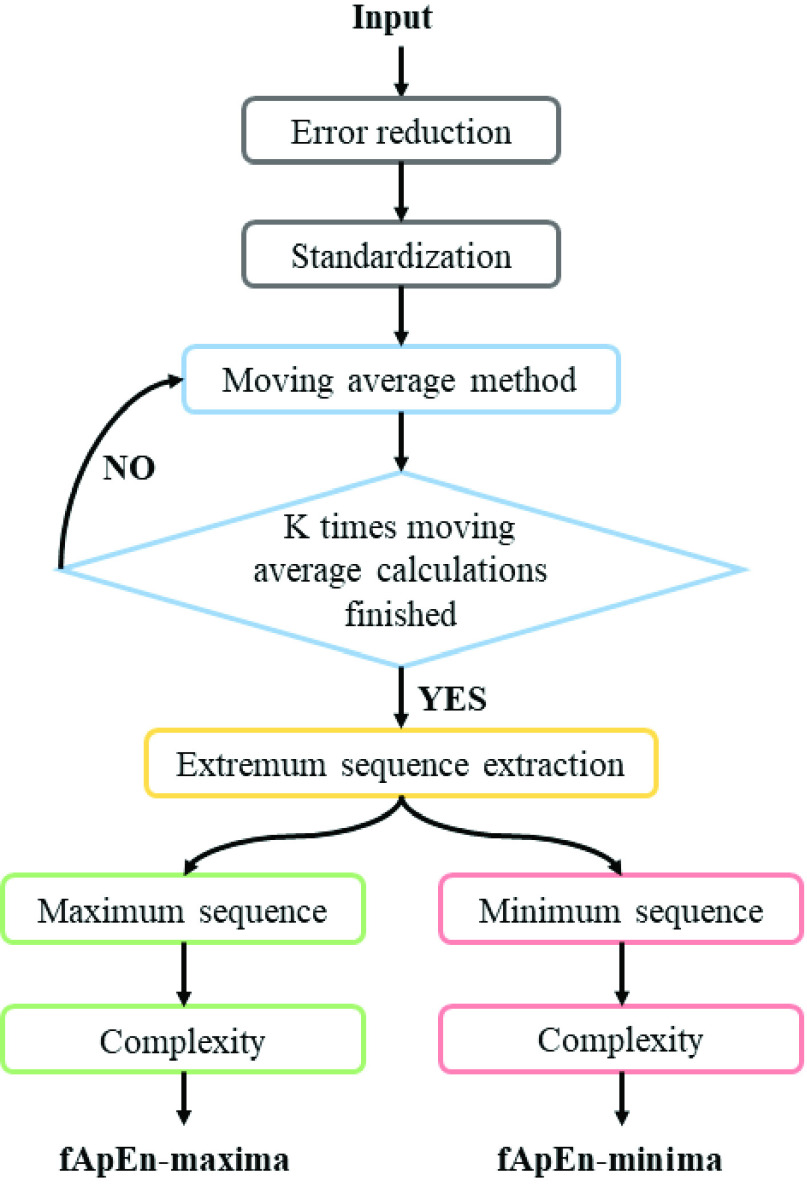
Scheme of multiple moving average analysis.

The extremum sequences can reflect the tension limits of the ANS, but it cannot be extracted directly from the RRs due to the small partial fluctuation of the RRs. Therefore, the RRs were filtered by the multiple moving average method first, and then the extremum sequences were extracted from them. Next, fApEn of the minima (fApEn-minima) and fApEn of the maxima (fApEn-maxima) were obtained by applying fApEn to calculate the complexity of the minimum and maximum sequences. The calculation method of the indices is shown in Fig. 2. First of all, the gross errors of the RRs were removed by the Pauta criterion [55]. And the Z-score method was used to standardize the RRs. Secondly, in order to extract the extremum sequences accurately, the multiple moving average method was used to reduce the local slight fluctuation of the RRs. Finally, fApEn-minima and fApEn-maxima were calculated to measure the complexity of extremum sequences.

**FIGURE 3.**
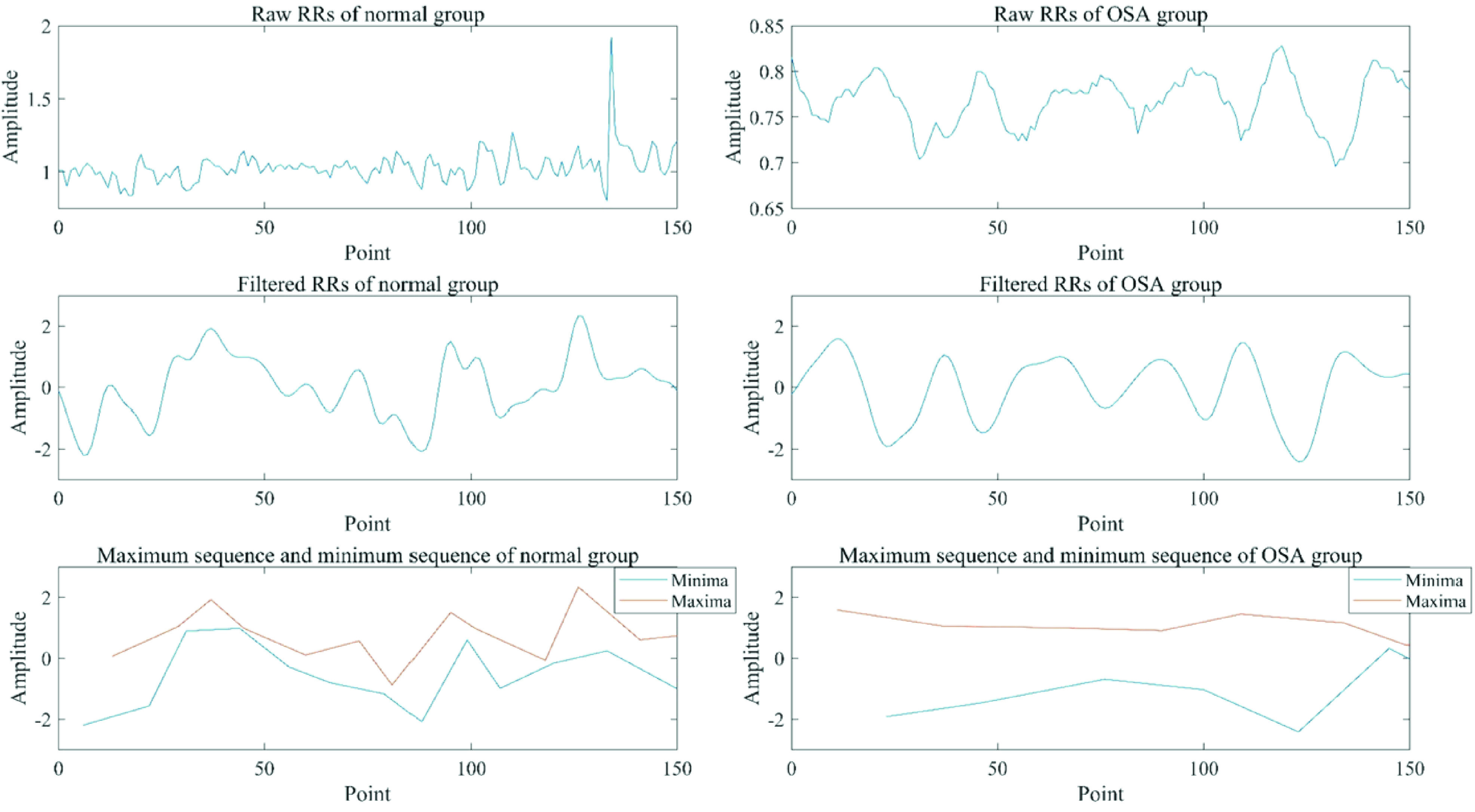
The comparison of the procedure diagrams of extremum sequences between the normal and OSA groups.

The raw RRs were divided into 1-minute non-repeated sequence subsets. First, since gross errors of the RRs can greatly affect the performance of the extremum sequences reflecting ANS tension limits, it is necessary to further remove them by Pauta criterion [55]. Then, the RRs were standardized. The details of the method are as described here:

The multiple moving average method can effectively reduce the small partial fluctuation of the sequence, with which the filtered sequence $O_{k}$ was obtained to reflect the general trend. Then the maximum sequence $U$ and the minimum sequence $V$ can be extracted from $O_{k}$. The details of the method are described here:

The comparisons of the raw RRs, the filtered RRs and the extremum sequences between the normal and OSA groups are shown in Fig. 3. The changes of amplitudes in the minimum and the maximum sequences of the OSA group are smaller than those of the normal group.

fApEn can effectively and robustly measure the complexity of the discrete sequence. The calculation details of it [56] are described here:

The self-matching was not eliminated in this study, which means $d_{ij}^{c}=0(i=j)$ in algorithm 3. In the calculation of fApEn, the parameters $m$, $n$ and $r$ were selected as 2, 2, and 0.25 times of the standard deviation of the input, respectively [56], [57], [58].

Algorithm 3 **(Fuzzy Approximate Entropy, fApEn)**
Input: Initialize the following parameters.
1) the non-stationary time sequence $X\{x_{1},x_{2},\ldots,x_{n} \}$;
2) the parameters $m$, $n$, $r$;
Output: the index $fApEn$
**for** $c=m$; $c\le m+1$; $c++$ **do**
**for** $i=1$; $i\le n-c+1$; $i++$ **do**
**for** $j=1$; $j\le c$; $j++$ **do**
$y_{i,j} =x_{i+j-1} -\frac {1}{c}\sum \limits _{k=0}^{c-1} {x_{i+k}} $
**end for**
**end for**
**for** $i=1$; $i\le n-c+1$; $i++$ **do**
**for** $j=1$; $j\le n-c+1$; $j++$ **do**
$d_{i,j}^{c} =\max \limits _{k=1,2,\ldots,c} \{\left |{ {y_{i,k} -y_{j,k}} }\right |\}$
$\mu _{i,j}^{c} =\exp \left [{ {-(d_{i,j}^{c} /r)^{n}} }\right]$
**end for**
**end for**
$S^{c}=\frac {1}{n-c+1}\sum \limits _{i=1}^{n-c+1} {\left({\frac {1}{n-c+1}\sum \limits _{j=1}^{n-c+1} {\mu _{i,j}^{c}} }\right)} $
**end for**
$fApEn=\ln S^{m}-\ln S^{m+1}$

According the algorithm 1–3, we can get the indices of Emma-fApEn.

Algorithm 1 **(Error Reduction and Standardization)**
Input: Initialize the following parameters.
1) the RRs $X\{X_{1},X_{2},\ldots,X_{n} \}$;
2) the point of $X_{i} \{x_{i,1},x_{i,2},\ldots,x_{i,m} \}$;
Output: the new sequence $Y\{Y_{1},Y_{2},\ldots,Y_{n} \}$
**for** $i=1$; $i\le n$; $i++$ **do**
calculate the average of $X_{i} $: $\overline {x_{i}} $
calculate the standard deviation of $X_{i} $: $s_{i} $
**for** $j=1$; $j\le m$; $j++$ **do**
**if** $\left |{ {x_{i,j} -\overline {x_{i}}} }\right |>3s_{i} $ **then**
delete $x_{i,j} $
**end if**
**end for**
$Y_{i} =(X_{i} -\overline {x_{i}})/s_{i} $
**end for**

Algorithm 2 **(Multiple Moving Average Extract the Extremum Sequence)**
Input: Initialize the following parameters.
1) the one-dimensional sequence $Q\{q_{1},q_{2},\ldots,q_{N} \}=\{Y_{1},Y_{2},\ldots,Y_{n} \}$;
2) the repeat counter $K$;
3) the interval length $M$;
Output: the maximum sequence $U$and the minimum sequence $V$
calculate the average length of $Y$: $L$
**for** $i=1$; $i\le K$; $i++$ **do**
**for** $j=1$; $j\le N-i\times M+i$; $j++$ **do**
$p_{j} =\frac {1}{M}\sum \limits _{k=j}^{j+M-1} {q_{k}} $
**end for**
**for** $j=1$; $j\le (N-i\times M+i)/L+1$; $j++$ **do**
build the one-dimensional sequence $P_{j} \{p_{(j-1)\times L+1},p_{(j-1)\times L+2},\ldots,p_{\min \{j\times L,N-i\times M+i\}} \}$
calculate the standard deviation of $P_{j} $: $s_{j} $
calculate the average of $P_{j} $: $\overline {p_{j}} $
$F_{j} =(P_{j} -\overline {p_{j}})/s_{j} $
**end for**
build the one-dimensional sequence $Q_{i} \{F_{1},F_{2},\ldots,F_{(N-i\times M+i)/L+1} \}$
**end for**
extract the maximum sequence $U$ and the minimum sequence $V$ from $O_{k}$

### C. Classifier

**FIGURE 4.**
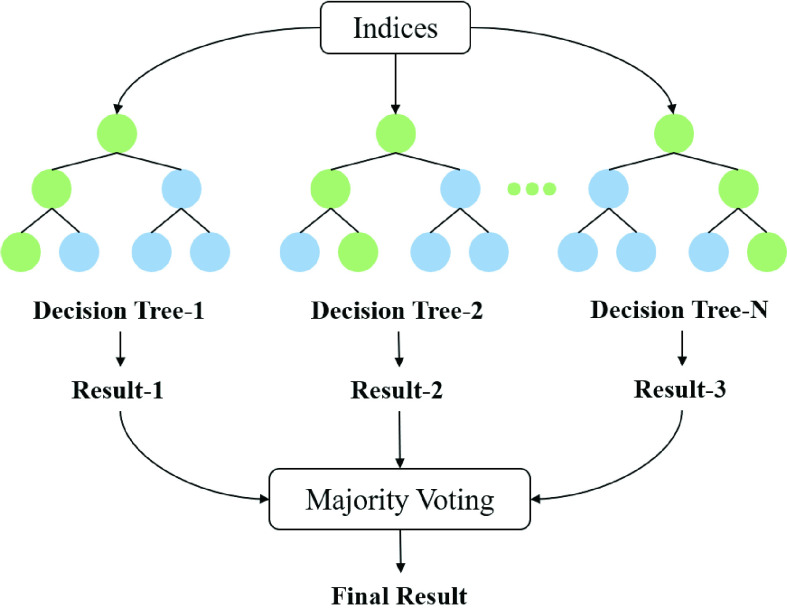
The structure of random forest.

In order to improve the detection performance of the indices and verify the complementarity of them, the random forest (RF) classifier was adopted for OSA and severe OSA screening. RF is composed of tree classifiers. As shown in Fig. 4, each tree classifier is generated by a random vector and exists independently of the input vector. In this study, Mean, LH, fApEn-minima and fApEn-maxima were used as the initial input of classifier. In the output part, the trees obtain the classification results of input vectors by voting the most popular category in units. RF used in this study uses randomly selected individual features or combinations of features to grow a tree in each node. Each decision tree is grown according to a random parameter. The classification results of all decision trees are integrated through the ensemble learning method to determine the final classification result.

The design of decision tree needs proper attribute selection measure and pruning method. Most methods choose attribute assignment quality measures to construct decision trees. Gini index, the most commonly used attribute selection measure in decision tree induction, is used to measure the total variance across two OSA classes. It is defined as:

\begin{align*} {Gini}_{N,M}=&p_{N} \left ({m }\right)\times \left [{ {1-p_{N} \left ({m }\right)} }\right] \\&+\,p_{M} \left ({m }\right)\times \left [{ {1-p_{M} \left ({m }\right)} }\right] \tag{7}\\ {Gini}_{M,S}=&p_{M} \left ({m }\right)\times \left [{ {1-p_{M} \left ({m }\right)} }\right] \\&+\,p_{S} \left ({m }\right)\times \left [{ {1-p_{S} \left ({m }\right)} }\right]\tag{8}\end{align*}

In (7), ${p}_{N} \left ({{m} }\right)$ represents the proportion of observation of the ${m}$ th node in the tree belonging to the normal and mild/moderate OSA groups; in (8), ${p}_{M} \left ({m }\right)$ represents the proportion of observation of the ${m}$ th node in the tree belonging to the mild/moderate OSA and severe OSA groups.

## III. Results

### A. Significance Analysis of Different Disease States

The different disease states analysis results, including the time-frequency domain indices and the multiple moving averages indices, are listed in Table 1. In the time-frequency domain indices, RMSSD could only distinguish the normal group from the mild/moderate OSA group. There were significant differences between the normal group and the other two groups in Mean and LH. As for the multiple moving averages indices, fApEn-minima and fApEn-maxima had outstanding performance. There were significant differences between the severe OSA group and the other two groups.

**TABLE 1:** Time-Frequency Domain and Multiple Moving Averages of HRV Indices for the Different Disease States During Sleep

Indices		N	M	S	P value		
					N&M	N&S	M&S
Time domain	Mean	0.987±0.111	0.850±0.095	0.883±0.090	<0.001***	0.001**	0.326
	SDNN	0.101±0.061	0.058±0.020	0.097±0.048	0.014*	0.759	0.020*
	RMSSD	0.106±0.108	0.036±0.017	0.067±0.050	0.006**	0.067	0.187
Frequency domain	LF	0.004±0.005	0.001±0.001	0.009±0.024	0.661	0.279	0.154
	HF	0.004±0.005	0.001±0.001	0.008±0.027	0.653	0.455	0.255
	LH	1.863±0.576	4.332±2.236	4.899±2.285	<0.001***	<0.001***	0.368
Multiple moving averages	fApEn-minima	1.995±0.016	1.974±0.049	1.803±0.169	0.598	<0.001***	<0.001***
	fApEn-maxima	1.993±0.019	1.941±0.069	1.832±0.130	0.111	<0.001***	0.001**

Data are expressed as mean ± standard deviation. N: normal group; M: mild/moderate OSA group; S: severe OSA group; P value: significance of difference. *, **, *** represents statistically significant difference P<0.05, P<0.01, P<0.001, respectively.

**FIGURE 5.**
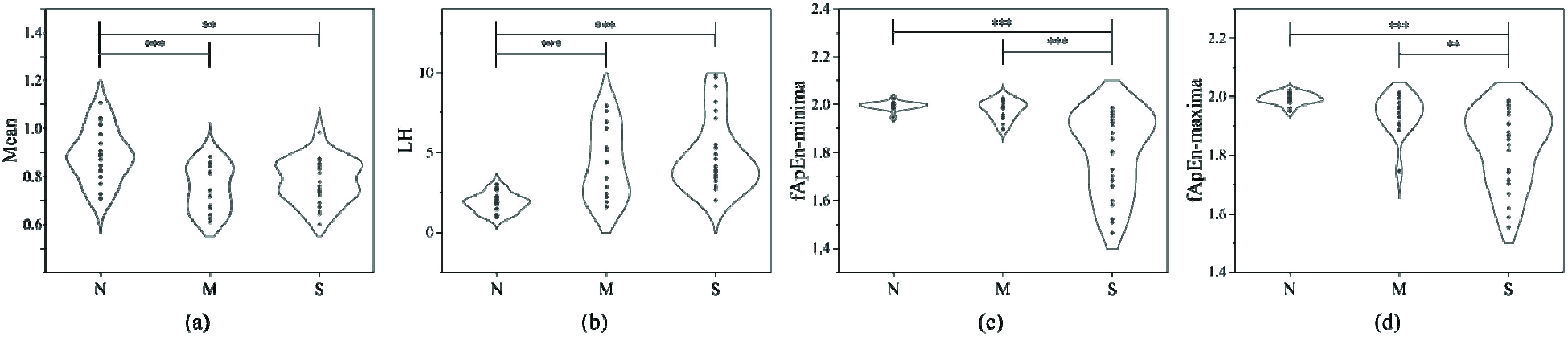
The comparison of Mean (a), LH (b), fApEn-minima (c) and fApEn-maxima (d) for the different disease states. N: normal group; M: mild/moderate OSA group; S: severe OSA group; *, **, *** represents statistically significant difference P<0.05, P<0.01, P<0.001, respectively.

The monotonicity analysis results of Mean, LH, fApEn-minima and fApEn-maxima in the different disease states are shown in the Fig. 5. There was a monotonically increasing trend of LH from the normal group to the mild/moderate OSA group to the severe OSA group, and monotonically decreasing trends of fApEn-minima and fApEn-maxima from the normal group to the mild/moderate OSA group to the severe OSA group. The variation patterns of Mean, LH, fApEn-minima and fApEn-maxima were statistically significant, demonstrating their effectiveness in the analysis of OSA severity.

### B. Correlation Analysis of the Useful Indices

**FIGURE 6.**
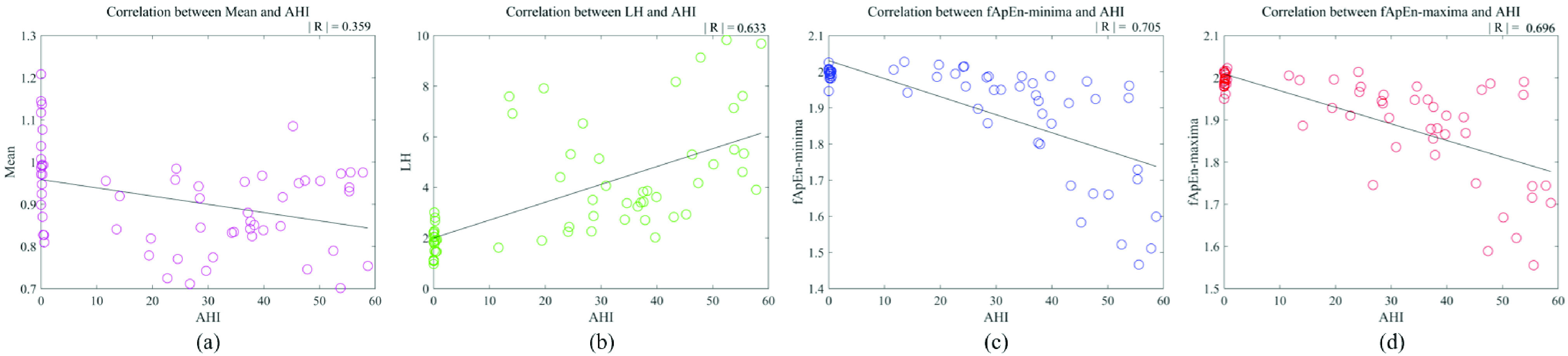
The correlative analysis of Mean (a), LH (b) fApEn-minima (c) and fApEn-maxima (d).

In this study, we analyzed the correlation of Mean, LH, fApEn-minima and fApEn-maxima with AHI. As shown in Fig. 6, Mean, LH, fApEn-minima and fApEn-maxima had a correlation ( $\left |{ R }\right |\ge 0.3$) with AHI. Moreover, LH, fApEn-minima and fApEn-maxima were significantly associated with AHI. The correlation coefficient of Mean ( $\left |{ R }\right |=0.359$) and LH ( $\left |{ R }\right |=0.633$) were lower than that of fApEn-maxima ( $\left |{ R }\right |=0.696$) and fApEn-minima ( $\left |{ R }\right |=0.705$). The above results indicate that fApEn-minima and fApEn-maxima have a higher correlation with AHI. Therefore, the relationships between the nonlinear indices and AHI are more significant.

### C. Parameter Optimization

In the calculation of Emma-fApEn, the multiple moving average method was used to reduce the interference of fine fluctuations in the RRs to the general trend. The moving average method reduced the interference while losing some information contained in the RRs, so the repeat counter $K$ and the interval length $M$ had an important impact on the results.

**FIGURE 7.**
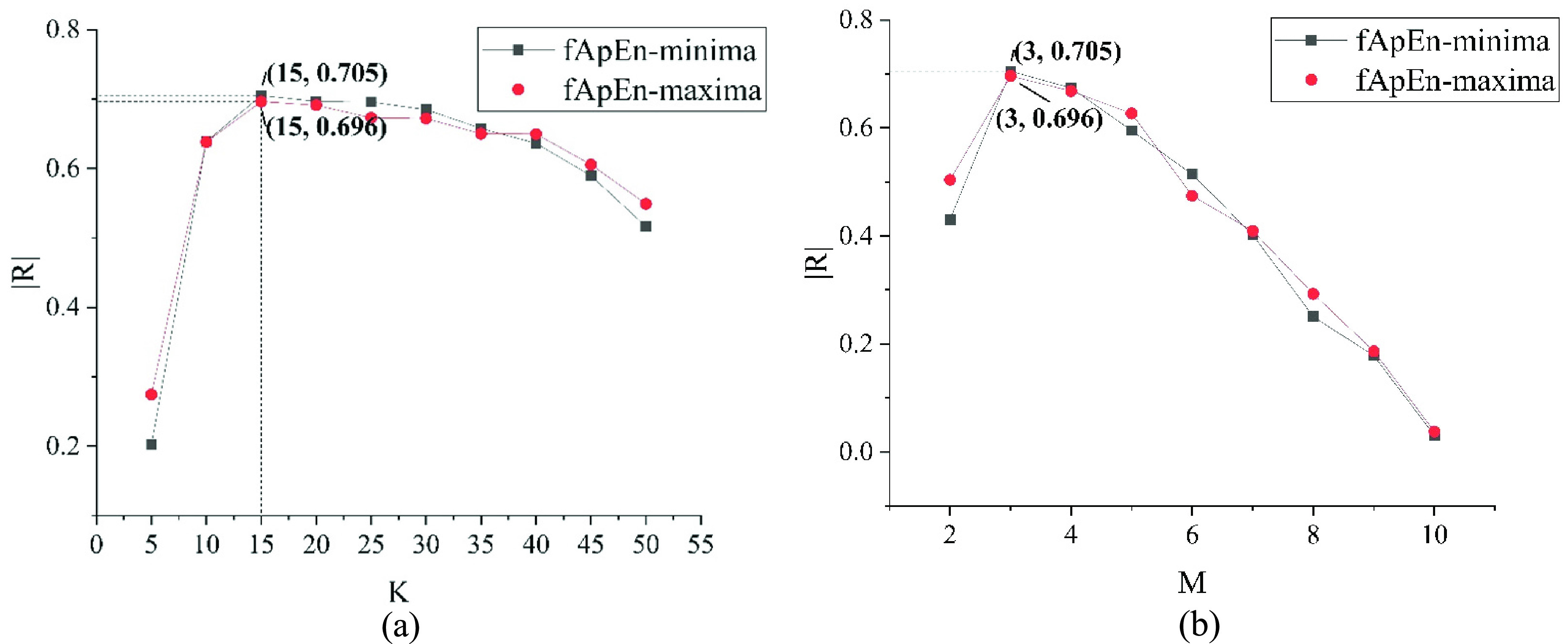
(a) Analysis of relationships between $\left |{ R }\right |$ and $K$ about fApEn-minima and fApEn-maxima. (b) Analysis of relationships between $\left |{ R }\right |$ and $M$ about fApEn-minima and fApEn-maxima. $\left |{ R }\right |$: the absolute value of the correlation coefficient between fApEn-minima or fApEn-maxima and AHI; $K$: the times of moving average method; $M$: the sliding interval scale.

The relationships between the absolute value of the correlation coefficient $\left |{ R }\right |$ and $K$ about fApEn-minima and fApEn-maxima when $M\textrm {=3}$ are shown in Fig. 7(a). It was obvious that fApEn-minima and fApEn-maxima had the highest correlation with AHI when $K\textrm {=15}$. The relationships between $\left |{ R }\right |$ and $M$ about fApEn-minima and fApEn-maxima when $K=15$ are shown in Fig. 7(b). It could be seen that the correlations between fApEn-minima and fApEn-maxima and AHI were the highest when $M=3$. In conclusion, fApEn-minima and fApEn-maxima performed better when we chose $K=15$ and $M=3$.

### D. OSA and Severe OSA Screening

The above analysis and the results in Table 1 show that Mean and LH have significant differences between the normal group and the OSA groups, and fApEn-minima and fApEn-maxima can significantly distinguish the severe OSA group from other two groups. These results indicate that Mean and LH have better detection performance in detecting OSA patients from normal people, and fApEn-minima and fApEn-maxima can effectively screen severe OSA patients from other people. Therefore, Mean, LH, fApEn-minima and fApEn-maxima were selected in this study for multi-indices OSA screening. Multiple machine learning classifiers were used to perform multi-indices detection, combining the advantages of the above four HRV indices to detect OSA and severe OSA patients.

**FIGURE 8.**
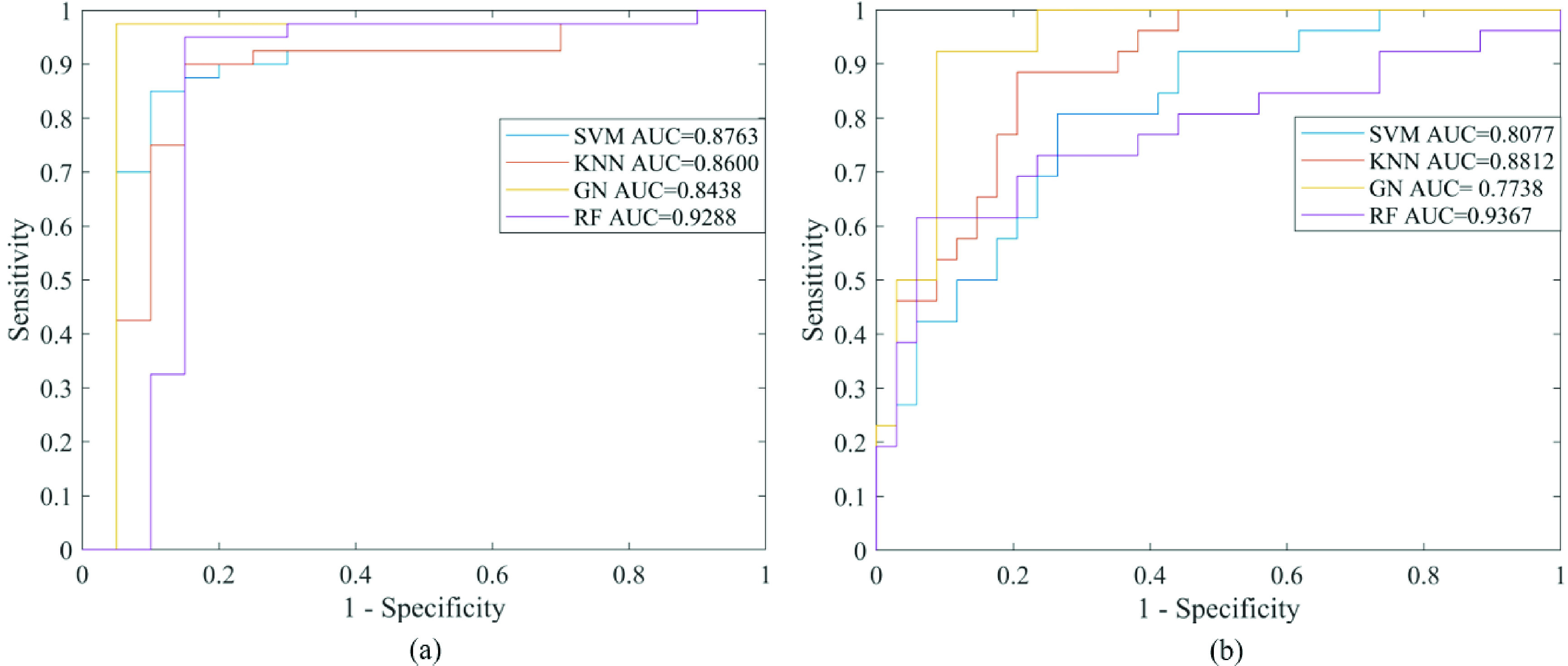
(a) The ROC curves of the machine learning methods to distinguish the normal group from the mild/moderate OSA and severe OSA groups. (b) The ROC curves of the machine learning methods to distinguish the severe OSA group from the normal and mild/moderate OSA groups.

Compared with other classifiers, we utilized random forest (RF) to implement the classification of the subjects based on two-fold cross-validation. The classification performance of multiple machine learning classifiers is shown in Table 2. As shown in Fig 8, the corresponding ROC curve of RF classifier achieves the best performance, and the AUC of RF classifier is obviously higher than others. The classification performance of RF classifier reached 96.67% accuracy to distinguish the normal group from the other two groups and 91.67% accuracy to distinguish the severe OSA group from the other two groups. They provided the highest accuracy and a good balance between sensitivity and specificity. Moreover, RF achieved the best AUC in OSA and severe OSA detection. The above results indicate that using RF classifier in the detection of OSA and severe OSA is significantly better than others. Therefore, RF classifier is chosen to achieve the OSA screening.

**TABLE 2:** Classification Performance of Three Classifiers (2-Fold Cross-Validation and Sequential Backward Selection)

Classifiers	N & non-N			S & non-S		
	Acc(%)	Spe(%)	Sen(%)	Acc(%)	Spe(%)	Sen(%)
SVM	86.67	89.90	84.96	76.67	74.31	81.55
KNN	88.33	85.35	90.23	83.33	80.21	89.29
GN	90.00	86.36	92.36	80.00	94.10	61.31
RF	96.67	95.45	97.62	91.67	91.32	92.86

N: normal group; non-N: mild/moderate OSA and severe OSA groups; S: severe OSA group; non-S: normal and mild/moderate OSA groups; Acc: accuracy; Sen: sensitivity; Spe: specificity; SVM: support vector machine; KNN: k-nearest neighbor; GN: gaussian naive-bayes; RF: random forest.

## IV. Discussion

### A. Comparison and Summary

The performance of the time-frequency domain indices was also verified by previous studies. The decreases of SDNN and RMSSD in OSA patients reported by Roche *et al.* [37] were consistent with our results. Lack of monotonicity with the degree of disease may be one of the reasons for their significance. Gula *et al.* [38] presented that LH was the most useful indicator for OSA detection, whose screening performance has been verified in our work. Although LH was a reliable index for screening OSA patients, there was no significant difference in LH between the mild/moderate OSA group and the severe OSA group. Therefore, LH was unable to distinguish different severities of OSA, which was in line with the findings of related studies [38], [45], [59]. Using HRV with the same database, our result was better than others. Haitham *et al.* presented that the sample entropy of HRV in OSA patients has the advantage of anti-noise, and the accuracy in OSA screening reached 70.3% [42]. Sliding trend fuzzy approximate entropy (SlTr-fApEn) was based on empirical mode decomposition method [56]. The accuracy of OSA screening increased to 85.0%. Li *et al.* reported that variance delay fuzzy approximate entropy (VD_fApEn) provides a method for ANS fluctuation analysis in OSA patients and OSA severity analysis, which showed an improved accuracy (90.0%) [45]. In this study, Emma-fApEn had a higher accuracy 96.67% in OSA screening and 91.67% in severe OSA screening. Therefore, Emma-fApEn is considered as a valid indicator for severity analysis in OSA screening.

Significance analysis showed that Mean and LH were effective indices for OSA screening. The results of the significance analysis also displayed that these time-frequency domain indices cannot discriminate the different severities of the disease. fApEn-minima and fApEn-maxima were significantly different between the severe OSA group and the other groups, which manifested their potential for differentiating severe OSA patients. Through correlation analysis, fApEn-minima ( $R=-0.705$) and fApEn-maxima ( $R=-0.696$) were negatively correlated with AHI, while LH ( $R=-0.633$) was positively correlated. As shown in Fig. 5, these three indices all showed great monotonicity with the OSA severity, while Mean not.

Machine learning methods were used to screen OSA and severe OSA, and the results showed that the accuracy of multi-indices OSA screening reached 96.67% by RF classifier, and the accuracy of severe OSA screening reached 91.67%. Compared with other classifiers, RF can better process high-dimensional data in the face of multi-indices processing and does not need feature screening.

**FIGURE 9.**
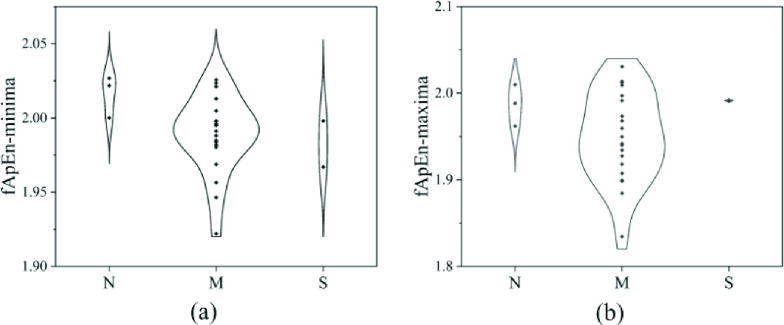
The comparison of fApEn-minima (a) and fApEn-maxima (b) for the different disease states. N: normal group; M: mild/moderate OSA group; S: severe OSA group.

Validation was implemented on St. Vincent’s University Hospital / University College Dublin Sleep Apnea Database downloaded from https://archive.physionet.org/pn3/ucddb/, which contains ECG recordings from 25 subjects. We extracted and corrected RRs from the database, and calculated the time-frequency domain indices and multiple moving average indices of the RRs. As shown in Table 3, fApEn-maxima and fApEn-minima have a higher correlation ( $\left |{ R }\right |\ge 0.2$) than other indices, whose violin plots are shown in Fig. 9. fApEn-minima was statistically significant ( $R=-0.538$), demonstrating the relationship between the nonlinear indices and AHI is more significant.

**TABLE 3:** Time-Frequency Domain and Multiple Moving Averages of HRV Indices for the R and P Value During Sleep Using Ucddb Database

Indices		R	P value
Time domain	Mean	−0.089	0.673
	SDNN	<0.001	0.998
	RMSSD	−0.009	0.967
Frequency domain	LF	−0.080	0.706
	HF	−0.112	0.594
	LH	0.138	0.511
Multiple moving averages	fApEn-minima	−0.538	0.005
	fApEn-maxima	−0.229	0.271

R: correlation coefficient; P value: significance of difference.

### B. Method Motivation Analysis

Moving average method is a simple low-pass filtering method, which can reduce the fluctuation of data and make data evener. Multiple moving average method can effectively obtain the general trend of the sequence by taking the moving average of it for many times. The general trend of the RRs is related to the general changes of ANS tension. The variation of the extrema of the RRs reflects fluctuations of ANS tension limits. Moreover, the multiple moving average method can prevent the selection of inappropriate extrema due to the small partial fluctuation of the RRs. Therefore, multiple moving average method was used to filter the RRs to obtain the general trend of them in this study. Since moving average method can change the mean and standard deviation of the sequence, it is necessary to normalize the RRs after each use of moving average method, which improves the robustness of the model.

Compared with approximate entropy and sample entropy, fApEn is less affected by calculated parameters. fApEn has a good robustness to noise, which can evaluate the complexity of biomedical signals more accurately [60]. The influence of self-matching on entropy is highly related to the length of sequence [61]. The lengths of extremum sequences are affected by the RRs fluctuations, and the fluctuations for normal controls are larger than for OSA patients in most cases. As a result, the extremum sequences of normal people obtained by multiple moving average method is longer than those of OSA patients. By comparison, it was found that the entropy obtained by self-matching was more correlated with AHI. Therefore, self-matching was not eliminated in the calculation of fApEn.

The repeat counter ${K}$ and the interval length $M$ of the moving average method have an essential impact on the calculation of Emma-fApEn. They directly affect the evaluation performance of extremum sequence complexity on ANS tension limits. The correlations between fApEn-minima, fApEn-maxima and AHI were the highest when we chose ${K}=15$ and $M=3$. Therefore, Emma-fApEn is able to better evaluate ANS tension limits under ${K}=15$ and $M=3$.

Machine learning methods were used to combine the advantages of multiple effective indices. The performance of different classifiers was compared. Screening results (Table 2) and ROC diagram (Fig. 8) show that RF classifier had the best performance in OSA screening and severe OSA detection. The accuracy of RF was higher than that of SVM, KNN and GN, while the sensitivity and specificity also reached a good balance. RF method combines all decision trees together, which not only improves the accuracy of classification and regression [62], but also is suitable for nonlinear analysis. Previous studies also indicated that RF classifier showed better detection performance to distinguish different severities of OSA [46], [47], [48]. Wrong predictions are made only if more than half of the base classifiers are wrong. RF is relatively stable. Even if a new data point appears in the database, which will only affect one decision tree. It’s difficult to affect all decision trees [62], [64]. Furthermore, each decision tree is built by random attribute selections and random samples from the original training set [63]. Outliers caused by noise in the samples will reduce the accuracy of the corresponding decision tree. The introduction of two types of randomnesses allows outliers to affect some decision trees instead of all the decision trees in a random forest. The forest is a vote of all decision trees, which is less affected by the decision trees with low accuracy. So RF does not overfit and has strong anti-noise performance. Unbiased estimation of generalization error can be realized while balancing errors, which enables the method to obtain the strong generalization ability [64].

### C. Physical Interpretation

Sudden fluctuations happen in ANS when sleep apnea occurs. The imbalance between the sympathetic and vagus nerve system can lead to ANS dysfunction [36]. HRV is proved to be an effective indicator to evaluate ANS function, and its reduction is one of the features of OSA occurrence [34]. As verified in this study, multiple classical time domain indices can be used to represent HRV changes [37]. Low frequency power reflects sympathetic tension, and high frequency power reflects parasympathetic tension. As their ratio, LH can reflect the balance between parasympathetic tone and sympathetic tone.

However, time-frequency domain analysis is linear and more suitable for stationary signal, while the process of regulating heart rate through ANS is nonlinear and non-stationary. In contrast, non-linear indices of HRV are better to analyze the complex dynamics of the heartbeat [41]. Therefore, nonlinear methods are usually used to analyze HRV [40]. In this study, multiple moving average method was used to weaken the small partial fluctuations of the RRs to obtain the general trend. The general trend of the RRs reflected the changes of physiological sympathetic tension during sleep. The extremum sequences in the general trend can reflect the changes of the relative tension limits of the sympathetic nerve. As shown in Fig. 3, the periodicity of the general trend of the RRs in OSA patients was obvious, and the fluctuation ranges of maximum and minimum sequences were significantly slighter than that of normal subjects. These results indicate that sympathetic tension in OSA patients enters a nearly periodic change when sleep apnea occurs. The spectrum of HRV is narrowed and the high frequency component decreases, which reduces the variation in the maxima and minima. Moreover, it reduces the ability of OSA patients to adapt to external changes [65]. These may be related to decreased tension and activity of ANS in OSA patients.

The sympathetic nervous system is enhanced and the parasympathetic nervous system is weakened when OSA occurs. Therefore, the low frequency component of HRV increases and the high frequency component decreases, resulting in the periodic fluctuation of HRV. The periodic fluctuations are manifested as the uniform fluctuation of HRV, which lead to the decrease of amplitude variation of the extremum sequences of HRV [66]. The periodic fluctuations of HRV basically do not occur in normal people, and the mild/moderate OSA patients have slight periodic fluctuations of HRV. In contrast, the increased periodic fluctuations and the reduced complexity of extremum sequences of HRV are more significant in the severe OSA patients. Moreover, traditional methods were to analyze the entire sequence of HRV. But we tended more to use the extremum sequences analysis of HRV. As the severity of OSA increased, the extremum sequences changed greatly, which leads to significant decreases in the complexity of fluctuations in ANS. The decreased tension and activity of ANS are more significant in severe OSA group. Therefore, non-linear indices of HRV we used in the study are more effective to screen severe OSA group than traditional metrics.

There are some limitations in this study. First, extremum sequences are greatly affected by the noise in the signal. Denoising method is important when obtaining extremum sequences. The data must conform to independent identically normal distribution [67], but the actual conditions do not generally meet this requirement. Therefore, the algorithm used in this study may not remove gross errors well. Second, there are differences in age and BMI among different groups in the data set we used. Individual differences are not taken into account in the method, which may affect the experimental results. Finally, potential cardiovascular diseases are not mentioned in this study, which may have influenced the HRV analysis. In the future studies, we will adopt more effective methods to improve the experimental method and verify the reliability of indices in more experimental data.

## V. Conclusion

In this study, Emma-fApEn was proposed as a HRV nonlinear analysis method for OSA research. Mean, LH, fApEn-minima and fApEn-maxima were used to analyze the features of HRV. The results showed that Mean and LH could significantly distinguish OSA patients from normal people. fApEn-minima and fApEn-maxima could significantly distinguish severe OSA patients from other people. Combined with the above four indices, RF was selected as the best classifier and achieved OSA screening with 96.67% accuracy as well as severe OSA screening with 91.67% accuracy. Therefore, the combination of Mean, LH, fApEn-minima and fApEn-maxima screening has an advantage over traditional single-indicator screening in analyzing HRV features and differentiating OSA severities. Moreover, this method also provides a new clinical reference for the diagnosis of OSA.
